# Examining racial and ethnic trends and differences in annual healthcare expenditures among a nationally representative sample of adults with arthritis from 2008 to 2016

**DOI:** 10.1186/s12913-020-05395-z

**Published:** 2020-06-12

**Authors:** Antoinette L. Spector, Sneha Nagavally, Aprill Z. Dawson, Rebekah J. Walker, Leonard E. Egede

**Affiliations:** 1 Department of Epidemiology, Institute for Health and Equity, Medical College of Wisconsin, 8701 Watertown Plank Rd., Milwaukee, WI 53226 USA; 2 Center for Advancing Population Science, Medical College of Wisconsin, 8701 Watertown Plank Rd., Milwaukee, WI 53226 USA; 3 Department of Medicine, Division of General Internal Medicine, Medical College of Wisconsin, 8701 Watertown Plank Rd., Milwaukee, WI 53226 USA

**Keywords:** Arthritis, Race, Ethnicity, Healthcare expenditures, Medical expenditure panel survey

## Abstract

**Background:**

Disparities in health care utilization and outcomes for racial and ethnic minorities with arthritis are well-established. However, there is a paucity of research on racial and ethnic differences in healthcare expenditures and if this relationship has changed over time. Our objectives were to: 1) examine trends in annual healthcare expenditures for adults with arthritis by race and ethnicity, and 2) determine if racial and ethnic differences in annual healthcare expenditures were independent of other factors such as healthcare access and functional disability.

**Methods:**

We used the Medical Expenditures Panel Survey (2008–2016) to examine trends in annual healthcare expenditures within and between racial and ethnic groups with arthritis (*n* = 227,663). A two-part model was used to estimate the marginal differences in expenditures by race and ethnicity after adjusting for relevant covariates, including the impact of healthcare access.

**Results:**

Between 2008 and 2016, there were no significant changes in unadjusted healthcare expenditures within any of the racial and ethnic groups, but the trend among non-Hispanic whites did differ significantly from Hispanics and Other. In fully adjusted analysis, mean annual expenditures for non-Hispanic whites was $946, $939, and $1178 more than non-Hispanic blacks, Hispanics, and Other, respectively (*p* < .001). Healthcare access also independently explained expenditure differences in this population with adults who delayed care spending significantly more ($2629) versus those who went without care spending significantly less (−$1591).

**Conclusions:**

Race and ethnicity are independent drivers of healthcare expenditures among adults with arthritis independent of healthcare access and functional disability. This underscores the need for ongoing research on the factors that influence persistent racial and ethnic differences in this population.

## Background

Arthritis (osteoarthritis and rheumatoid arthritis) impacts more than 65 million people in the United States, is the leading cause of disability among adults, and results in annual health care expenditures that exceed $600 billion annually [1–4]. Common medical services used to manage the joint pain and stiffness that accompany arthritis include office visits with primary care and specialty physicians, prescription drugs, physical and occupational therapy, and surgical procedures [4, 5]. Thus, it is estimated that adults with arthritis spend $1000 more annually on health care, compared to those without this condition [4, 6].

Although people with arthritis are engaging with the healthcare environment regularly, racial and ethnic minorities use fewer health care services than non-Hispanic Whites (NHW) [4, 7, 8]. Non-Hispanic Blacks (NHB) and Hispanics are less likely than non-Hispanic Whites to have rehabilitation services, see an orthopedic specialist, undergo a total joint arthroplasty, or utilize complementary and alternative medicine (CAM) to manage their condition [8–18]. Further, Hispanic and Asian-Americans with arthritis are approximately three and four times more likely, respectively, to have forgone any type of treatment compared to NHW [11].

There are several explanations posited for these differences including a reduced preference for, less access to, and worse outcomes with conventional medical services. Some studies have found NHB to prefer nontraditional treatment methods, such as natural remedies and spirituality, and to expect worse outcomes with surgical management [10, 18–21]. There is also evidence that racial and ethnic minorities experience greater barriers to utilizing traditional services to treat their arthritis, such as lower socioeconomic status and higher rates of being publicly insured or uninsured [11, 22, 23]. This disparity in access could result in greater unmet health care need and worse health status, as well as increased health care costs over time [10, 14, 24–27].

Notwithstanding our awareness of disparities in health care utilization by racial and ethnic minorities, there has been little attention paid to how this has impacted people with arthritis over time. While Raval and Vyas examined trends in healthcare expenditures among people with arthritis and found less utilization by racial and ethnic minorities across service categories between 2008 and 2014, they did not analyze if there were differences in trends between racial and ethnic groups [4]. Moreover, there have been changes in the health care landscape that may have impacted the magnitude of racial and ethnic differences. The full implementation of the Patient Protection and Affordable Care Act (ACA), in 2014, has led to increased health care utilization by racial and ethnic minorities and reduced their likelihood of forgoing or delaying care [28–30]. Therefore, the purpose of this study was twofold. We first wanted to examine trends in annual healthcare expenditures for adults with arthritis by race and ethnicity and identify if there were significant differences within and between groups. Second, we wanted to identify if there were significant racial and ethnic differences in annual healthcare expenditures among people with arthritis, independent of other factors such as healthcare access and functional disability.

## Methods

### Data source and sample

We retrospectively examined an overall sample of 227,663 individuals age 18 years and older and a subsample of 53,058 individuals with a self-reported diagnosis of arthritis between 2008 and 2016 using the full-year household consolidated data files of the Medical Expenditure Panel Survey (MEPS) [31]. MEPS is an annual national household survey that derives estimates of healthcare utilization, health status, and health insurance coverage from a nationally representative sample of the civilian noninstitutionalized U.S. population [32]. A complex, stratified sampling strategy is used to obtain a unique and representative sample for each year that the survey is administered. By combining data from a series of years, this analysis provides a series of cross-sectional snapshots overtime. To provide three points in time to assess trends while also maintaining sufficient sample size in the individual time periods, we divided the total sample between three different time periods (2008–2010, 2011–2013, 2014–2016). The 2008–2010 sample had a total of 72,415 individuals with 16,685 reporting an arthritis diagnosis. The 2011–2013 sample had a total of 79,160 individuals with 17,843 reporting an arthritis diagnosis. The 2014–2016 sample had a total of 75,482 individuals with 18,530 reporting an arthritis diagnosis.

### Dependent variable

The dependent variable was total direct healthcare expenditures. This includes the sum of payments for medical services such as office-based visits, inpatient stays, emergency room visits, home healthcare visits, and prescription drug costs. Annual total expenditures account for an individual’s out-of-pocket expenses, payments made by public and private insurance providers, and other sources during the calendar year. Expenditure data is collected at the household level and through medical providers [32].

### Independent variable

The primary independent variable was race/ethnicity. We grouped the sample into four categories: non-Hispanic white (NHW), non-Hispanic black (NHB), Hispanic (HSP), and non-Hispanic other (OTH). OTH includes those who were non-Hispanic and identified as Asian, American Indian/Alaska Native, Native Hawaiian/Pacific Islander, or multiracial.

### Covariates

The remaining variables were included based on the Andersen and Newman Framework of Healthcare Utilization, and were categorized as predisposing, enabling and need factors [33, 34]. Andersen and Newman theorized that there are several individual determinants of healthcare utilization and provided a model to assist researchers in identifying relevant variables in their analysis [34]. Predisposing factors include characteristics that existed before the onset of illness and can be associated with differing patterns in service utilization [34]. Enabling factors are the resources available to an individual to allow them to obtain medical care [34]. Need factors are the perceived or evaluated presence of an illness that would provide a reason for an individual to obtain medical care [34]. Andersen and Newman provide suggestions of variables that could be included in each category and indicate that each variable and category are distinct enough to be included in multivariate analyses [34]. Additionally, the Andersen and Newman model has previously been used to identify relevant variables in MEPS among a population of individuals with arthritis [6].

#### Predisposing

Predisposing factors included age, sex, region, and metropolitan statistical area (MSA). Region was categorized as Northeast, Midwest, South, and West.

#### Enabling

Enabling factors were education, employment status, poverty level, insurance status, and access to medical care. Education was dichotomized as having less than a bachelor’s degree versus having at least a bachelor’s degree. Employment was divided into employed versus unemployed. Poverty level was categorized by the percentage of family income in relation to the federal poverty line into five income groups: high (> to 400%), middle (< 400% and > 200%), low (< 200 and > 125%), near poor (> 125% and > 100%), or poor (< 100%). Insurance status was grouped by those with any private insurance, only publicly insured, or uninsured. Access to medical care was determined in two ways. The first type was based on a yes or no response to a question asking if the individual delayed needed care during the calendar year. The second was based on a yes or no response to whether the individual went without needed medical care during the calendar year.

#### Need

Need factors were comorbidities, body mass index (BMI), self-reported joint pain, and needing help with activities of daily living (ADL) or instrumental activities of daily living (IADL). We summed the number of self-reported medical conditions (high blood pressure, heart disease, stroke, emphysema, chronic bronchitis, high cholesterol, cancer, diabetes) to create a comorbidity count. We categorized BMI into four groups including normal weight (18.5 to < 25), underweight (< 18.5), overweight (25 to < 30), and obese (30 or higher). Joint pain was included as a binary variable based on a response of either *yes* or *no*. For ADL and IADL, we dichotomized each variable based on whether a person responded either *yes* or *no* to needing help completing ADL (examples: feeding, bathing, dressing, toileting) or IADL (examples: using the telephone, paying bills, taking medications, preparing light meals, doing laundry, going shopping).

### Statistical analyses

We first calculated demographics of the 2008–2016 pooled sample using means, frequencies, and percentages. Second, we identified linear trends in mean annual healthcare expenditures and prevalence of arthritis by race and ethnicity in adults (18 years or older) with a self-reported diagnosis of arthritis between 2008 and 2016. We executed the Cochran Armitage test to identify significant differences between trend lines and unadjusted linear models to identify significant differences within a single trend line. Following Manning and Mullahy’s recommendation on handling healthcare expenditure data, we then used a two-part statistical model to assess the likelihood of a zero or positive healthcare expenditure and the spending differences among those who had greater than a zero amount of expenditure [35, 36]. This approach has been incorporated in previously published research using the MEPS dataset [37]. Using this method, we assessed both binomial and positive distributions of healthcare expenditures. To assess the likelihood of zero expenditure versus positive expenditure, we implemented a probit model followed by a gamma distributed and log link generalized linear model (GLM) to assess the differences among the population who had positive healthcare expenditures. The GLM gamma distribution and log link in the second part of the model was used to account for the skewness of the medical expenditure data by transforming medical expenditures into log scale. GLM gamma family and log link were chosen because of their ability to avoid bias associated with retransformation to raw scale, it is able to handle heteroscedasticity in non-negative data, and is a more flexible methodology that is able to relax normality requirements [38, 39]. For weighting the U.S. population, we implemented survey design packages which integrates sampling weights, stratum and psu clustering [40]. Finally, to calculate adjusted healthcare expenditures, we calculated average marginal effects using the post estimation commands. To confirm changes in expenditures over time were independent of inflation, we ran a second set of models adjusting expenditure by the Consumer Price Index. All analyses were performed using R and STATA v.15 [41]. A statistical significance of *p* < 0.05 was used for all analyses.

## Results

Table 1 shows the demographic characteristics of the sample. Among the total sample (*n* = 227,663) there was an overall arthritis prevalence of 23% (*n* = 53,058) between 2008 and 2016. The sample was predominantly female (54%) and NHW (43%), lived in the South (38%), and were either high (29%) or middle (30%) income. Most had some private insurance (57%), less than a bachelor’s degree (72%), and were employed (63%).

**Table 1:** Sample demographics by arthritis status in U.S. adults 2008–2016 (*n* = 227,663)

	Total n (%)	Arthritis n (%)	
		Yes	No
All	227,663 (100)	53,058 (23)	173,999 (76)
Age			
18–34	75,029 (33)	2912 (5)	71,980 (41)
35–44	40,841 (18)	5017 (9)	35,740 (21)
45–64	75,563 (33)	24,112 (45)	51,166 (29)
> =65	36,230 (16)	21,017 (40)	15,113 (9)
Sex			
Male	105,808 (46)	19,411 (37)	86,106 (49)
Female	121,855 (54)	33,647 (63)	87,893 (51)
Race/Ethnicity			
Hispanic	62,471 (27)	8637 (16)	53,725 (31)
Non-Hispanic White	97,981 (43)	29,303 (55)	68,364 (39)
Non-Hispanic Black	44,672 (20)	11,651 (22)	32,896 (19)
Other	22,539 (10)	3467 (7)	19,014 (11)
Region			
Northeast	36,212 (16)	8823 (17)	27,319 (16)
Midwest	43,470 (19)	11,581 (22)	31,765 (18)
South	86,081 (38)	21,319 (40)	64,527 (37)
West	61,900 (27)	11,335 (21)	50,388 (29)
Education			
Less than bachelor’s	142,906 (72)	35,022 (75)	108,762 (72)
Bachelor’s degree or more	54,868 (28)	11,652 (25)	43,163 (28)
Employment			
Employed	143,289 (63)	21,460 (41)	121,648 (70)
Not employed	83,402 (37)	31,482 (59)	51,778 (30)
Poverty Category			
High income [> = 400]	66,206 (29)	15,104 (28)	50,923 (29)
Middle Income [> = 200 & < 400]	68,321 (30)	15,025 (28)	53,099 (31)
Low Income [> = 125 & < 200]	37,626 (17)	8829 (17)	28,690 (16)
Near Poor [> = 100 & < 125]	13,566 (6)	3590 (7)	9946 (6)
Poor [< 100]	41,944 (18)	10,545 (20)	31,341 (18)
Insurance Status			
Any Private	129,900 (57)	28,239 (53)	101,543 (58)
Public only	52,15 (23)	20,200 (38)	32,552 (19)
Uninsured	44,948 (20)	4619 (9)	39,904 (23)
Year			
2008–2010	72,625 (32)	16,685 (31)	55,730 (32)
2011–2013	79,362 (35)	17,843 (34)	61,317 (35)
2014–2016	75,676 (33)	18,530 (35)	56,952 (33)

Table 2 shows the arthritis prevalence overall and for each of the four race/ethnicity categories. NHW had the highest prevalence of arthritis during each of the study periods (29–32%), followed by NHB (26–27%), OTH (15–16%), and HSP (13–15%). There were no significant differences in arthritis prevalence within or between any of the racial and ethnic groups during the study period.

**Table 2:** Arthritis prevalence by race and ethnicity 2008–2016^a^ (*n* = 227,663)

Race/Ethnicity	2008–2010			2011–2013			2014–2016		
	Arthritis (n)	No Arthritis (n)	Prevalence	Arthritis (n)	No Arthritis (n)	Prevalence	Arthritis (n)	No Arthritis (n)	Prevalence
Total	16,685	55,730	30%	17,843	61,317	29%	18,530	56,952	33%
NHW	9769	23,899	29%	9653	23,436	29%	9881	21,029	32%
NHB	3539	10,233	26%	4093	11,956	26%	4019	10,707	27%
HSP	2362	15,965	13%	2941	19,229	13%	3334	18,531	15%
OTH	1015	5633	15%	1156	6696	15%	1296	6685	16%

*NHW* Non-Hispanic white, *NHB* Non-Hispanic black, *HSP* Hispanic, *OTH* Non-Hispanic other

^a^No significant changes in prevalence over time within or between groups

**Fig. 1:**
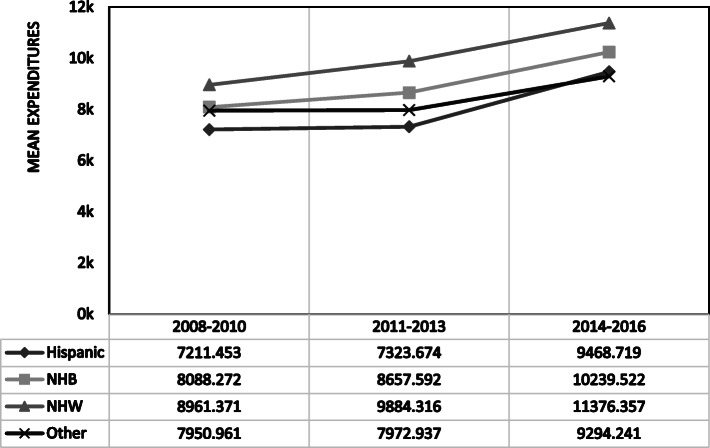
Mean annual expenditures among adults with arthritis by race and ethnicity 2008–2016

Figure 1 shows mean expenditures by race and ethnicity during each time period (2008–2010, 2011–2013, and 2014–2016). NHW with arthritis had the highest mean annual expenditure during each of the study periods. There were increases in expenditures over time in each of the racial and ethnic groups. NHW spent on average $8961 per year between 2008 and 2010 with an increase to $11,376 per year by 2014–2016. For NHB, mean annual expenditures were $8088 per year between 2008 and 2010 and increased to $10,240 per year between 2014 and 2016. OTH spent $7951 on average per year between 2008 and 2010 with an increase to $9294 per year in 2014–2016. HSP had the lowest expenditures of the racial and ethnic groups but also increased from $7211 per year in 2008–2010 to $9469 per year in 2014–2016. While an increase in mean annual expenditures was observed across all groups from 2008 to 2010 to the 2014–2016 time period, none of the linear cost trends reached the level of significance within any of the groups. However, the Cochran-Armitage test found that there were significant differences in mean expenditure trends between racial and ethnic groups. The trends in mean expenditures were significantly different for HSP (*p* = 0.04) and OTH (*p* < 0.001) compared to NHW. There were no significant differences in mean expenditure trends between NHW and NHB.

Findings from the adjusted analyses are shown in Table 3. The adjusted model reflects marginal differences in mean annual expenditures among adults with arthritis after adjusting for predisposing, enabling, and need factors. In this model, each of the racial and ethnic minority groups had significantly less mean annual expenditures than NHW. On average, NHW had $939 more in expenditures than HSP (*p* < 0.001), $946 more than NHB (*p* < 0.001), and $1178 more than those in the OTH group (*p* < 0.001). Based on the 95% confidence interval around the adjusted mean annual expenditures, there were no significant differences between the racial and ethnic minority groups. After adjustment, differences in mean expenditures were also observed over time for adults with arthritis. Compared to 2008–2010, mean expenditures increased by $750 (*p* < 0.01) for 2011–2013 and $900 (*p* < 0.001) for 2014–2016. After accounting for inflation using the Consumer Price Index, there was a marginal increase in expenditures at the *p* = 0.09 level by $406 for 2011–2013 and $269 (*p* = 0.23) for 2014–2016.

**Table 3:** Two-part regression model: adjusted marginal differences in healthcare expenditures among U.S. adults with arthritis by race and ethnicity 2008–2016 (*n* = 53,058)

	Marginal difference	95% Confidence interval	*P*-value
Race/Ethnicity			
Non-Hispanic White (Reference)	–	–	
Non-Hispanic Black	-$946	-$1355 to -$537	***
Hispanic	-$939	-$480 to -$1397	***
Other	-$1178	-$1713 to -$644	***
Year			
2008–2010 (Reference)	–	–	
2011–2013	$758	$341 to $1176	**
2014–2016	$897	$51 to $1281	***
*Predisposing*			
Age	-$24.36	-$39 to -$9	***
Sex			
Male (Reference)	–	–	
Female	$362	$22 to $701	*
Region			
Northeast (Reference)	–	–	
Midwest	-$86	-$830 to $99	
South	-$451	-$883 to -$20	
West	-$45	-$524 to $434	
*Enabling*			
Education			
< Bachelor’s Degree (Reference)	–	–	
> = Bachelor’s degree	$1119	$708 to $1530	
Employment			
Employed (Reference)	–	–	
Unemployed	$2305	$1839 to $2771	
Poverty Category [family income to poverty line %]			
High income [> = 400] (Reference)	–	–	
Middle Income [> = 200 & < 400]	-$851	-$1295 to -$407	**
Low Income [> = 125 & < 200]	-$845	-$1415 to -$275	*
Near Poor [> = 100 & < 125]	-$1035	-$1609 to -$460	**
Poor [< 100]	-$966	-$1518 to -$415	**
Insurance Status			
Any Private (Reference)	–	–	
Public only	-$830	-$1214 to -$445	***
Uninsured	-$3937	-$4500 to -$3373	***
Access to Care			
Able to get care (Reference)	–	–	
Unable to get care	-$1591	-$2152 to -$1030	***
No delays in receiving care (Reference)	–	–	
Delayed in receiving care	$2629	$1905 to $3353	***
*Need*			
Comorbidity Count (0–8)	$1575	$1427 to $1722	***
Functional Limitations			
Did not need help with ADL (Reference)	–	–	
Needed help with ADL	$5241	$4184 to $6298	***
Did not need help with IADL (Reference)	–	–	
Needed help with IADL	$3829	$3118 to $4540	***
BMI			
Normal weight (Reference)	–	–	
Under weight	$1407	$205 to $2609	*
Overweight	$91	-$358 to $540	
Obese	$452	$40 to $863	
Joint Pain			
No Joint Pain (Reference)	–	–	
Had joint pain	$1349	$914 to $1785	***

*p < 0.05; **p < 0.01; ***p < 0.001

There were several other factors that independently explained mean expenditure differences among adults with arthritis in the fully adjusted model (Table 3). Those who went without needed care had expenditures that were $1591 less than those who reported that they were able to obtain needed medical care during the calendar year (*p* < 0.001). Adults with arthritis who reported delaying needed medical care had expenditures that were $2629 more on average than those who received timely care (*p* < 0.001). Additionally, those who were uninsured had significantly lower expenditures (−$3937; *p* < 0.001) than those with any private insurance. Needing help with ADL or IADL resulted in higher annual expenditures of about $5200 and $3800, respectively (*p* < 0.001). For each additional comorbidity reported by those with arthritis, there was a $1575 increase in mean annual expenditures during the study period (*p* < 0.001).

## Discussion

The goals of our study were to examine trends in annual healthcare expenditures among adults with arthritis by race and ethnicity, and to identify factors that explained differences in costs within this population using nationally representative data from 2008 to 2016. We found that the overall arthritis prevalence remained stable during the study period overall and within each of the racial and ethnic groups. We observed a significant increase in average annual expenditures in each of the later cohorts compared to 2008 through 2010. After adjusting for inflation to 2019 dollars using the Consumer Price Index marginally, statistically significant increases in expenditures remained for 2011–2013 and 2014–2016 compared to 2008–2010 indicating this increase was not due to inflation. We also found that expenditure trends in Hispanic and Other racial and ethnic minorities with arthritis were significantly different than NHW during the study period with mean annual expenditures that were between $900 and $1200 less than NHW independent of other access and functional ability factors incorporated into our model. Although predisposing, enabling, and need factors were all identified as key drivers of medical expenditures in adults with arthritis, they did not negate the racial and ethnic differences.

Our findings are consistent with previous work on racial and ethnic differences in healthcare expenditures and builds on previous research in two important ways. First, we identified that trends in healthcare expenditures among NHW with arthritis were significantly different than HSP and OTH between 2008 and 2016. Williams et al. previously found that individuals with arthritis and joint pain had over $1600 higher expenditures annually despite additional differences by race and functional limitations [6]. Among adults with arthritis, Raval and Vyas found that unadjusted annual healthcare expenditures were consistently lower among all racial and ethnic minorities compared to NHW during each year of their study (2008–2014) [4]. Taken together, these findings suggest an ongoing need to study racial and ethnic differences in healthcare expenditures among individuals with arthritis and how these differences are changing over time. Given the dynamic nature of the healthcare environment, analyses are also warranted to examine the specific factors that are influencing differing trends in healthcare expenditures within this population.

The second unique contribution of this analysis is identifying that healthcare expenditures among racial and ethnic minorities remained significantly lower than NHW after adjusting for predisposing, enabling, and need factors, including healthcare access and activities of daily living. Although previous research has not specifically examined cost, researchers have posited that healthcare access, socioeconomic status, and education might explain differences in healthcare utilization by racial and ethnic minorities with arthritis [14, 24]. Our study provides evidence that these factors do not fully account for differences in mean annual expenditures. Health beliefs are considered an important aspect of healthcare utilization and may also impact expenditures [33, 34]. Previous research has found that racial and ethnic minorities with arthritis prefer complementary and alternative therapies to conventional medical care, which would generally be excluded from healthcare expenditures [10, 18, 19, 21]. The utilization of and expenses for complementary and alternative therapies could be an important part of understanding racial and ethnic differences in expenditures among adults with arthritis.

We did find notable differences in annual expenditures among adults with arthritis who encountered barriers to medical care. A study by Molina et al. concluded that delays in treatment resulted in worse clinical outcomes in a community sample of adults with rheumatoid arthritis [42]. Our study is the first, to our knowledge, to quantify the direct cost of delaying needed care in a nationally representative sample of adults with arthritis. We found that those that delayed care spent $2629 more on average than those receiving timely care. Additional research is needed to examine the circumstances surrounding the delayed care and could provide opportunities for intervention that reduce the financial burden and improve clinical outcomes in this population.

Despite the novelty of our findings, this study did have some limitations. Our sample was identified from a cohort of participants who self-reported an arthritis diagnosis. Self-report data is subject to recall bias and may have impacted our prevalence estimates. However, the arthritis prevalence identified by MEPS has been shown to agree with estimates in other national surveys [43]. Our study was also limited to the data available in MEPS. Although we were able to incorporate several predisposing, enabling, and need factors, MEPS does not include data on clinical outcomes, such as disease activity, or health beliefs. Additionally, utilization of many complementary and alternative therapies is not accounted for in MEPS.

## Conclusions

In conclusion, our study found that trends in annual expenditures for non-Hispanic whites were similar to non-Hispanic blacks between 2008 and 2016, but significantly different from Hispanics and other racial minority groups. Additionally, after accounting for healthcare access and functional disability factors, racial and ethnic minorities had significantly lower mean annual expenditures than non-Hispanic whites and delaying needed care was associated with substantially higher costs among adults with arthritis. Our findings provide additional support to the persistence of racial and ethnic differences in healthcare engagement among this population beyond access concerns, as well as the financial burden of delaying needed care. Future research on adults with arthritis should aim to quantify the impact of health beliefs, and the utilization of complementary and alternative therapies, on racial and ethnic differences in healthcare expenditures.

## Data Availability

The datasets analyzed during the current study are available in the Agency for Healthcare Research and Quality: Medical Expenditure Panel Survey repository, https://meps.ahrq.gov/mepsweb/data_stats/download_data_files.jsp [31].

## Abbreviations

ACA: Patient Protection and Affordable Care Act
ADL: Activities of daily living
BMI: Body mass index
CAM: Complementary and alternative medicine
HSP: Hispanic
IADL: Instrumental activities of daily living
MEPS: Medical Expenditure Panel Survey
MSA: Metropolitan statistical area
NHB: Non-Hispanic black
NHW: Non-Hispanic white
OTH: Non-Hispanic other
